# Mining of Adverse Event Signals Associated with Fluticasone Furoate/Umeclidinium/Vilanterol Triple Therapy: A Post-Marketing Analysis Based on FAERS

**DOI:** 10.3390/pharmacy13060178

**Published:** 2025-12-10

**Authors:** Jiajun Chen, Ying Qiao, Gaoxing Qiao, Xiaocan Jia, Jicun Zhu

**Affiliations:** 1 Department of Medical Services, The First Affiliated Hospital of Zhengzhou University, Zhengzhou 450052, China; fccchenjiajun@zzu.edu.cn; 2 Department of Epidemiology and Biostatistics, College of Public Health, Zhengzhou University, Zhengzhou 450001, China; clyerqy@163.com; 3 Department of Pharmacy, The First Affiliated Hospital of Zhengzhou University, Zhengzhou 450052, China; fccqiaogx@zzu.edu.cn

**Keywords:** COPD, fluticasone furoate, umeclidinium, vilanterol, adverse event, FAERS

## Abstract

Chronic obstructive pulmonary disease (COPD) is a major global health burden. The fluticasone furoate (FF)/umeclidinium (UMEC)/vilanterol (VI) triple therapy provides new treatment, but its long-term real-world safety lacks evidence. A post-marketing analysis used the FAERS database to identify adverse event (AE) signals for FF/UMEC/VI. Disproportionality methods including reporting odds ratio (ROR), proportional reporting ratio (PRR), information component (IC), and empirical Bayesian geometric mean (EBGM), were applied to detect AE signals, focusing on reports from third quarter (Q3) 2019 to Q3 2024. Among 16,238 reports listing FF/UMEC/VI as primary suspect, significant AE signals occurred in ‘injury, poisoning and procedural complications’ (*n* = 9067, ROR 2.46, PRR 2.08, IC 1.06, EBGM 2.08), and ‘respiratory, thoracic and mediastinal disorders’ (*n* = 6567, ROR 4.87, PRR 4.15, IC 2.05, EBGM 4.13). A total of 196 significantly disproportionate preferred terms (PTs) were identified, including previously undocumented AEs such as chronic eosinophilic rhinosinusitis, dysphonia, and vocal cord dysfunction. This post-marketing safety study revealed significant signals for dysphonia and vocal cord dysfunction associated with FF/UMEC/VI, suggesting that clinicians should remain vigilant for these events.

## 1. Introduction

Chronic obstructive pulmonary disease (COPD) is a prevalent, chronic inflammatory airway disorder characterized by persistent airflow limitation and dyspnea [1,2]. Its global health burden has risen by 25.7% over the past three decades, generating substantial medical and socioeconomic costs [3,4]. Although long-acting bronchodilators and inhaled corticosteroids (ICS) mitigate symptoms, they do not fully address pulmonary micro-thrombosis, cardiovascular complications, or other systemic sequelae [5,6,7]. Consequently, identifying therapeutic regimens with both superior efficacy and an acceptable safety profile remains a clinical priority.

To effectively control COPD symptoms, the combination therapy of long-acting muscarinic antagonists (LAMAs) and long-acting beta2 agonists (LABAs) has emerged as a crucial strategy in the treatment of COPD [8]. However, there remains room for improvement in its efficacy. Triple therapy with fluticasone furoate (FF), umeclidinium (UMEC), and vilanterol (VI) is a novel inhaled therapeutic regimen that integrates ICS, LABA, and LAMA [9,10]. This combination has been demonstrated to significantly improve lung function and quality of life in COPD patients, owing to its comprehensive approach to airway inflammation control and bronchodilation [11,12,13]. Despite the FF/UMEC/VI triple therapy having shown significant efficacy in clinical trials, there is a lack of studies on its long-term safety and adverse events (AEs) in the real world.

The FDA Adverse Event Reporting System (FAERS) database, as a vital resource in pharmacovigilance research, offers a platform for the global scientific community to monitor and analyze drug-related AEs [14]. Pharmacovigilance studies utilizing spontaneous reporting systems like FAERS are critical for identifying rare or long-term safety signals not captured in clinical trials. We endeavor to mine and analyze post-marketing AE signals associated with FF/UMEC/VI triple therapy using the FAERS database. Signal detection through disproportionality analysis provides a validated methodology to screen potential drug-event associations in real-world populations. For FF/UMEC/VI—a triple-therapy agent—evaluating post-marketing safety is essential given its recent approval (2019) and limited real-world safety data beyond registration studies.

The aim of this study is to clarify the safety profile of this treatment regimen in real-world clinical settings, and mine AEs compared to the traditional FF/VI therapy. This analysis may improve our understanding of the safety of triple therapy across diverse patient populations, identify potential risk factors, and provide critical data to support ongoing clinical research and drug development initiatives.

## 2. Materials and Methods

### 2.1. Study Design and Data Sources

An observational, retrospective disproportionality analysis based on a case/non-case study design was conducted to investigate AEs associated with FF/UMEC/VI, utilizing data from the FAERS database. As FF/UMEC/VI received FDA approval on 18 November 2019, the analysis included all reports submitted to FAERS from the third quarter of 2019 (Q3) to the third quarter of 2024 (Q3).

### 2.2. Data Extraction

**Figure 1:**
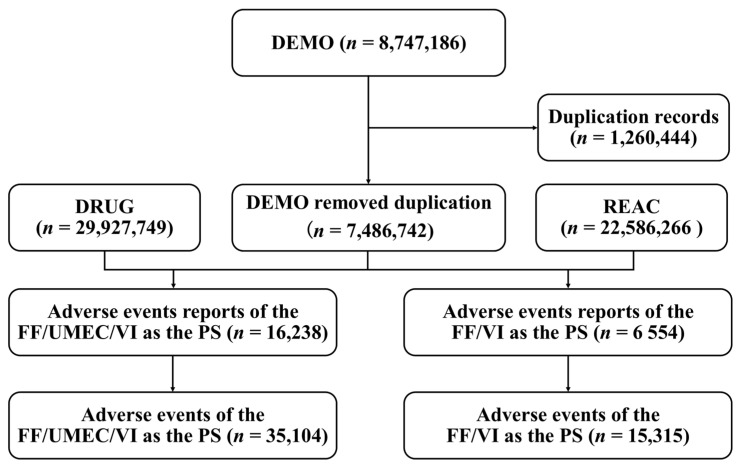
Flowchart of data collection process. FF, fluticasone furoate; UMEC, umeclidinium; VI, vilanterol; DEMO, Demographic/administrative file; DRUG, Drug information file; REAC, Reaction file; PS, primary suspect.

The FAERS data were obtained from the FDA’s official website at https://www.fda.gov/drugs/questions-and-answers-fdas-adverse-event-reporting-system-faers/fda-adverse-event-reporting-system-faers-public-dashboard (accessed on 17 December 2024). The database was searched for reports containing ‘Trelegy Ellipta’ (brand name) or ‘fluticasone furoate/umeclidinium/vilanterol’ (generic name) for the triple therapy, and ‘Breo Ellipta’ or ‘fluticasone furoate/vilanterol’ for FF/VI. To ensure the accuracy of the drug-AE associations, the analysis focused only on reports in which the target drugs were explicitly identified as the primary suspect (PS) drugs. To eliminate duplicate entries, we followed FDA guidelines by selecting the most recent FDA report date for duplicate Unique case version identifier [15], and, in cases where both the FDA report date and Unique case identifier were identical, the higher Unique case version identifier was retained. FAERS literature-derived cases were included in the dataset. Duplicates between literature and spontaneous reports were resolved using the same Unique case version identifier/FDA report date prioritization method. Detailed information, including patient characteristics (e.g., sex and age), reporting region, outcomes, and time-to-onset (TTO), was subsequently retrieved and analyzed. The workflow for FAERS data handling and analysis-covering data retrieval from core files (DEMO: Demographic/administrative file, DRUG: Drug information file, REAC: Reaction file)-is illustrated in Figure 1. All AEs were coded using the preferred terms (PT) and primary system organ class (SOC) classifications from the Medical Dictionary for Regulatory Activities (MedDRA, version 26.0, English).

### 2.3. Statistical Analysis

In this study, disproportionality methods, including reporting odds ratio (ROR), proportional reporting ratio (PRR), information component (IC), and empirical Bayesian geometric mean (EBGM), were simultaneously employed to detect AE signals based on a 2 × 2 contingency table [16]. A significant signal was defined as an association with at least three case reports and concurrently meeting the thresholds for all four algorithms: ROR (lower limit of the 95%CI > 1), PRR (≥2 with χ^2^ ≥ 4), IC (IC025 > 0), and EBGM (EBGM05 ≥ 2). Expected events were those already listed in the drug label. Signals that were not documented in the drug label were classified as unexpected signals, which may represent potential new safety concerns requiring further clinical evaluation to assess causality. Higher values in these measures indicate greater statistical disproportionality in reporting frequency, which may suggest a potential association between the drug and AE. Furthermore, we conducted a direct comparative analysis of AE profiles between FF/UMEC/VI and FF/VI to evaluate the specific safety impact of adding UMEC to the FF/VI dual therapy regimen. All data mining and statistical analyses were performed using SAS version 9.4 (SAS Institute Inc., Cary, NC, USA).

## 3. Results

### 3.1. Descriptive Analysis

**Figure 2:**
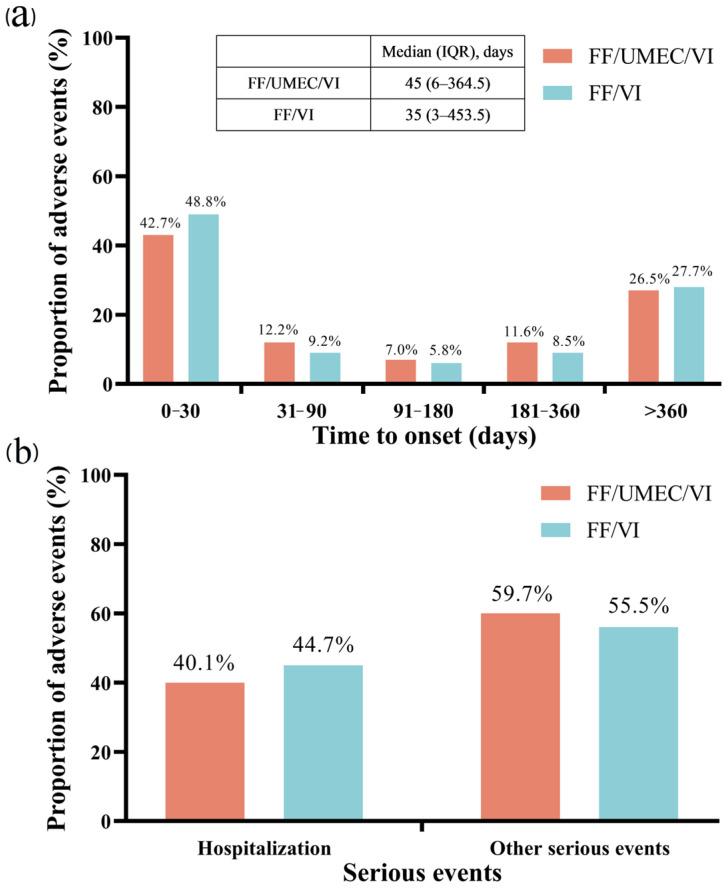
Time to onset of FF/UMEC/VI and FF/VI-related adverse events. (**a**) onset times of adverse events. (**b**) serious events. FF, fluticasone furoate; UME, umeclidinium; VI, vilanterol; IQR, interquartile range.

From 2019 (Q3) to 2024 (Q3), a total of 8,747,186 AE reports were recorded in the FAERS database. After removing duplicates, 16,238 reports were identified with FF/UMEC/VI as the primary suspected drug, while 6554 reports were associated with FF/VI (Table 1). The proportion of male patients exceeded that of female patients for both drugs under study (46.09% vs. 30.47% for FF/UMEC/VI, and 54.43% vs. 18.70% for FF/VI, respectively). The median age for patients associated with FF/UMEC/VI and FF/VI was 72 years (IQR: 65–79) and 70 years (IQR: 60–78), respectively. The median onset time for FF/UMEC/VI-related AEs was 45 days (IQR: 6–364.5), while that for FF/VI-related AEs was 35 days (IQR: 3–453.5) (Figure 2a). Among serious event outcomes, hospitalization rates were 40.1% and 41.7% with FF/UMEC/VI and FF/VI treatment, respectively (Figure 2b).

### 3.2. System Organ Class Disproportionality Analysis

In the disproportionate analysis of SOCs, we identified 27 organ systems that were involved in FF/UMEC/VI-induced AEs (Table 2 and Table S1). The significant SOCs that met three criteria were ‘injury, poisoning and procedural complications’ (*n* = 9067, ROR 2.46, PRR 2.08, IC 1.06, EBGM 2.08), ‘respiratory, thoracic and mediastinal disorders’ (*n* = 6567, ROR 4.87, PRR 4.15, IC 2.05, EBGM 4.13), and Product issues (*n* = 1915, ROR 2.98, PRR 2.87, IC 1.52, EBGM 2.86).

### 3.3. Preferred Terms Disproportionality Analysis

We further examined the PT signals and identified a total of 196 significantly disproportionate PTs that met the criteria of all four algorithms for FF/UMEC/VI. In this study, these PTs were ranked by ROR, and the top 10 are presented in Table 3, while the other significant PTs ranked by frequency were detailed in Table S2. Notably, we identified strong AE signals that were not documented in the drug labels, including chronic eosinophilic rhinosinusitis (*n* = 3, ROR = 96.32, PRR = 96.31), dysphonia (*n* = 639, ROR = 21.07, PRR = 20.71), and vocal cord dysfunction (*n* = 9, ROR = 16.71, PRR = 16.30), all of which were all unexpected AEs. In addition, the significant AEs included bronchospasm paradoxical (*n* = 5, ROR = 50.97, PRR = 50.96), candida infection (*n* = 564, ROR = 54.86, PRR = 24.68), and decreased urine flow (*n* = 17, ROR = 19.68, PRR = 19.67), which were consistent with the drug instruction.

### 3.4. The Comparison Among FF/UMEC/VI and FF/VI

At the SOC level, comparative disproportionality analysis revealed significantly lower reporting of ‘injury, poisoning and procedural complications’ for FF/UMEC/VI versus FF/VI (*n* = 9607, ROR = 0.76, PRR = 0.82; Table S3). No significant difference was observed for ‘respiratory, thoracic and mediastinal disorders’ (*n* = 6567, ROR = 1.02, PRR = 1.02).

Table 4 lists the top 10 most significant AE signals based on PT levels when compared with FF/VI. In addition to decreased blood pressure (*n* = 18, ROR = 7.86, PRR = 7.85), other top significant AE signals were consistent with the drug’s prescribing information. These included urinary retention (*n* = 157, ROR = 8.60, PRR = 8.56), paradoxical bronchospasm (*n* = 93, ROR = 6.78, PRR = 6.76), and constipation (*n* = 84, ROR = 3.34, PRR = 3.33).

## 4. Discussion

Most studies have evaluated the association between individual drugs and AEs [17], whereas this study focused on the FF/UMEC/VI triple combination therapy. We found that among the 16,238 AEs reported after the market launch of the FF/UMEC/VI triple therapy in the FAERS database, the primary signals were ‘injury, poisoning and procedural complications’ and ‘respiratory, thoracic and mediastinal disorders’. The results were similar to the types of AEs observed in some clinical trials, further confirming the commonality of these AEs in the treatment with this class of drugs [18,19,20]. The FULFIL study showed that FF/UMEC/VI significantly improved FEV1 values and SGRQ scores, and reduced the rate of moderate/severe acute exacerbations compared to budesonide and formoterol [21]. The IMPACT trial indicated that FF/UMEC/VI was effective in reducing all-cause mortality compared with dual therapy drugs [22,23]. Although the FF/UMEC/VI triple therapy has higher drug costs, the overall cost-effectiveness was still superior to the budesonide/formoterol therapy due to the reduction in acute exacerbations and other related expenses [24]. These research findings provide important reference for health policy-makers.

Further analysis of the PT signals identified 196 significantly disproportionate PTs, including some strong AE signals that were not recorded in the drug labels, such as chronic eosinophilic rhinosinusitis, dysphonia, and vocal cord dysfunction, which may be related to the diversity and complexity of patient populations in the real world. Eosinophils have diverse multifaceted immunobiological characteristics, which exacerbate local inflammatory responses in the development of chronic eosinophilic rhinosinusitis [25]. Patients with an eosinophilic COPD phenotype exhibit a T2-high inflammation driven by cytokines like IL-5, IL-4, and IL-13, which promotes eosinophil recruitment and activation in both the bronchial and sinus mucosa. In this context, the effective control of lower airway symptoms by FF/UMEC/VI may bring pre-existing or concomitant chronic eosinophilic rhinosinusitis into sharper clinical focus. Most patients with chronic eosinophilic rhinosinusitis have high viscous nasal secretions, accompanied by olfactory dysfunction and multiple bilateral nasal polyps [26]. Voice disorders are the most common local side effects of ICS, which may be due to the cause of inflammation and candidiasis caused by the deposition of steroids on the laryngeal mucosa [27]. A case–control study showed that the use of steroid inhalers was associated with a 5.11-fold increased risk of dysphonia compared with the control group [28]. These findings suggest that we need to pay more attention to these potential risks when using the FF/UMEC/VI triple therapy in clinical practice. These potential safety signals require confirmation through well-designed studies before regulatory consideration [29]. If validated, their inclusion in risk management plans would optimize safety monitoring.

In addition, FF/UMEC/VI shows differences in the proportion of certain AEs compared to FF/VI, which may be related to its multiple mechanisms of action. FF is an inhaled corticosteroid with anti-inflammatory activity [30]. VI is a selective long-acting β2 receptor agonist that can relax bronchial smooth muscle and relieve bronchospasm [31]. UMEC is a long-acting muscarinic receptor antagonist that produces bronchodilation by competitively inhibiting the binding of acetylcholine to the M3 muscarinic receptors on the airway smooth muscle [32]. It is important to note that the signal for decreased blood pressure appears pharmacologically inconsistent with the known profiles of UMEC and VI. Therefore, this statistical signal should not be interpreted as a direct adverse drug effect. It is more likely attributable to confounding factors inherent in the real-world COPD population, such as the widespread use of concomitant antihypertensive medications and the high burden of underlying cardiovascular diseases, which can lead to events being reported in temporal association with drug use without a causal relationship. The longer median TTO for AEs with FF/UMEC/VI (45 days) versus FF/VI (35 days) is likely attributable to channeling bias, as triple therapy is typically reserved for patients with more severe COPD, which may alter AE reporting patterns.

Through the synergistic action of the above mechanisms, the FF/UMEC/VI triple therapy can significantly improve lung function in COPD patients, reduce the frequency of acute exacerbations, and improve quality of life [9,20]. Additionally, based on these mechanisms of action, FF/UMEC/VI has recently been used in the treatment of asthma with significant efficacy [33,34]. However, quantitative analysis shows specific increases in urinary retention (Absolute Risk Reduction, ARR = 2.8), constipation (ARR = 1.9), and dry mouth (ARR = 1.7), consistent with UMEC’s M3 muscarinic receptor antagonism that impairs smooth muscle contractility. Importantly, no significant risk elevation occurred for corticosteroid- or β2-agonist-related events (candidiasis and tremor), confirming these effects originate specifically from UMEC.

Although this study provides valuable information, there are also some limitations. Firstly, reporting bias and underreporting are inherent to spontaneous reporting systems; the database may over-represent serious or well-known events while missing a substantial number of actual cases, affecting the comprehensiveness of the data. Secondly, a critical limitation is the lack of denominator data; because the total number of patients treated is missing, we cannot calculate the incidence of adverse events and are limited to analyzing reporting proportions. Furthermore, the variable quality and completeness of clinical data precluded stratified analyses by patient age, sex, report seriousness, or specific comorbidities, which would have offered deeper insights. Consequently, we are unable to establish causality between AEs and drugs based on these data alone. Notably, no methods beyond disproportionality analysis were employed to assess causality or control for confounding factors, and the lack of detailed patient information (e.g., baseline health status and concomitant medication use) further complicates causal inference.

## 5. Conclusions

In summary, this study characterizes the real-world safety profile of FF/UMEC/VI triple therapy and detects novel pharmacovigilance signals requiring further validation. Clinicians need to not only pay attention to common AEs but also be alert to dysphonia and vocal cord dysfunction AE signals when using FF/UMEC/VI. These findings provide a basis for the rational use of drugs in clinical practice to offer data support for future research and drug regulation. Despite the significant therapeutic effects demonstrated in clinical trials, the safety of the treatment still needs to be closely monitored in practical applications.

## Figures and Tables

**Table 1:** Demographic and clinical characteristics of patients treated with FF/UMEC/VI and FF/VI.

Characteristics	FF/UMEC/VI	FF/VI	*p* Value
Total	16,238	6554	
Sex, *n* (%)			<0.001
Female	4948 (30.47)	1881 (28.70)	
Male	7484 (46.09)	3895 (59.43)	
Missing	3806 (23.44)	778 (11.87)	
Age (year), *n* (%)			<0.001
<18	22 (0.14)	19 (0.29)	
18≤ and <65	887 (5.46)	590 (9.00)	
≥65	2847 (17.53)	1090 (16.63)	
Missing	12,482 (76.87)	4855 (74.08)	
Median (IQR)	72 (65, 79)	70 (60, 78)	
Reporting country, *n* (%)			<0.001
United States	14,636 (90.13)	6489 (99.01)	
Non-United States	1602 (9.87%)	65 (0.99)	
Reporter, *n* (%)			<0.001
Consumer	13,964 (86.00)	6085 (92.84)	
Health professional	786 (4.84)	162 (2.47)	
Physician	1001 (6.16)	163 (2.49)	
Pharmacist	444 (2.73)	131 (2.00)	
Missing	43 (0.26)	33 (0.50)	
Reporting year, *n* (%)			<0.001
2019	891 (5.49)	1185 (18.08)	
2020	2702 (16.64)	1299 (19.82)	
2021	2911 (17.93)	549 (8.38)	
2022	3479 (21.43)	954 (14.56)	
2023	3461 (21.31)	1890 (28.84)	
2024	2794 (17.21)	677 (10.33)	

Abbreviations: FF, fluticasone furoate; UMEC, umeclidinium; VI, vilanterol.

**Table 2:** FF/UMEC/VI AE signal strength vs. other drugs ranked by SOC frequency.

SOC	*n*	ROR (95% CI)	$\mathrm{PRR}\;(\chi^{2})$	IC (IC025)	EBGM (EBGM05)
Injury, poisoning and procedural complications	9067	2.46 (2.40, 2.52)	2.08 (5810.78)	1.06 (1.02)	2.08 (2.04)
Respiratory, thoracic and mediastinal disorders	6567	4.87 (4.74, 5.00)	4.15 (16,324.16)	2.05 (2.01)	4.13 (4.04)
General disorders and administration site conditions	5215	0.81 (0.79, 0.83)	0.84 (198.86)	0.26 (0.3)	0.84 (0.82)
Infections and infestations	2142	1.09 (1.04, 1.13)	1.08 (13.49)	0.11 (0.05)	1.08 (1.04)
Product issues	1915	2.98 (2.84, 3.12)	2.87 (2367.56)	1.52 (1.45)	2.86 (2.75)
Gastrointestinal disorders	1547	0.54 (0.51, 0.57)	0.56 (573.31)	0.83 (0.91)	0.56 (0.54)
Nervous system disorders	1439	0.54 (0.52, 0.57)	0.56 (526.82)	0.83 (0.91)	0.56 (0.54)
Surgical and medical procedures	1210	2.36 (2.23, 2.50)	2.31 (913.40)	1.21 (1.12)	2.31 (2.20)
Investigations	804	0.38 (0.35, 0.41)	0.39 (796.60)	1.34 (1.45)	0.39 (0.37)
Musculoskeletal and connective tissue disorders	776	0.42 (0.39, 0.45)	0.43 (611.29)	1.21 (1.32)	0.43 (0.41)
Eye disorders	666	0.97 (0.90, 1.05)	0.97 (0.45)	0.04 (0.15)	0.97 (0.91)
Cardiac disorders	649	0.95 (0.88, 1.02)	0.95 (1.86)	0.08 (0.19)	0.95 (0.89)
Psychiatric disorders	549	0.28 (0.26, 0.30)	0.29 (999.66)	1.78 (1.90)	0.29 (0.27)
Renal and urinary disorders	533	0.82 (0.75, 0.89)	0.82 (21.45)	0.29 (0.41)	0.82 (0.76)
Neoplasms benign, malignant and unspecified (incl cysts and polyps)	518	0.37 (0.34, 0.41)	0.38 (533.60)	1.38 (1.51)	0.38 (0.36)
Skin and subcutaneous tissue disorders	467	0.23 (0.21, 0.26)	0.24 (1156.84)	2.03 (2.17)	0.24 (0.23)
Vascular disorders	241	0.37 (0.32, 0.42)	0.37 (259.96)	1.42 (1.61)	0.37 (0.34)
Immune system disorders	193	0.47 (0.41, 0.55)	0.48 (112.15)	1.07 (1.28)	0.48 (0.42)
Social circumstances	190	1.13 (0.98, 1.31)	1.13 (2.99)	0.18 (0.03)	1.13 (1.01)
Metabolism and nutrition disorders	169	0.25 (0.21, 0.29)	0.25 (386.34)	2.00 (2.22)	0.25 (0.22)
Ear and labyrinth disorders	92	0.64 (0.52, 0.78)	0.64 (18.71)	0.64 (0.94)	0.64 (0.54)
Reproductive system and breast disorders	59	0.28 (0.22, 0.36)	0.28 (109.82)	1.83 (2.21)	0.28 (0.23)
Blood and lymphatic system disorders	46	0.07 (0.06, 0.10)	0.08 (525.00)	3.71 (4.14)	0.08 (0.06)
Hepatobiliary disorders	23	0.08 (0.05, 0.12)	0.08 (249.66)	3.66 (4.25)	0.08 (0.06)
Endocrine disorders	23	0.25 (0.16, 0.37)	0.25 (53.21)	2.02 (2.61)	0.25 (0.18)

Abbreviations: FF, fluticasone furoate; UMEC, umeclidinium; VI, vilanterol; SOC, system organ class; ROR, reporting odds ratio; CI, confidence interval; PRR, proportional reporting ratio; *χ*^2^, chi-squared; IC, information component; EBGM, empirical Bayesian geometric mean.

**Table 3:** Top 10 AE signal strength of FF/UMEC/VI vs. other drugs ranked by PT ROR.

SOC	PT	*n*	ROR (95% CI)	$\mathrm{PRR}\;(\chi^{2})$	IC (IC025)	EBGM (EBGM05)
Respiratory, thoracic and mediastinal disorders	Chronic eosinophilic rhinosinusitis	3	96.32 (28.62, 324.16)	96.31 (246.06)	6.39 (4.83)	83.88 (30.39)
	Bronchospasm paradoxical	5	50.97 (20.5, 126.71)	50.96 (226.89)	5.56 (4.34)	47.29 (22.07)
	Dysphonia	639	21.07 (19.46, 22.81)	20.71 (11,620.28)	4.33 (4.21)	20.09 (18.8)
	Vocal cord dysfunction	9	16.71 (8.62, 32.38)	16.70 (129.49)	4.03 (3.1)	16.3 (9.37)
	Paranasal sinus inflammation	4	16.36 (6.06, 44.14)	16.36 (56.25)	4 (2.69)	15.98 (6.96)
Injury, poisoning and procedural complications	Foreign body in mouth	5	48.65 (19.6, 120.76)	48.64 (216.89)	45.29 (21.16)	5.5 (4.27)
Infections and infestations	Oropharyngeal candidiasis	14	30.9 (18.07, 52.84)	30.89 (386.34)	29.52 (18.84)	4.88 (4.12)
	Candida infection	264	24.86 (21.97, 28.12)	24.68 (5777.46)	23.8 (21.47)	4.57 (4.39)
Gastrointestinal disorders	Coating in mouth	10	29.46 (15.63, 55.54)	29.45 (262.82)	4.82 (3.93)	28.21 (16.59)
Renal and urinary disorders	Urine flow decreased	17	19.68 (12.14, 31.89)	19.67 (292.27)	4.26 (3.57)	19.11 (12.76)

Abbreviations: FF, fluticasone furoate; UMEC, umeclidinium; VI, vilanterol; SOC, system organ class; PT, preferred terms; ROR, reporting odds ratio; CI, confidence interval; PRR, proportional reporting ratio; *χ*^2^, chi-squared; IC, information component; EBGM, empirical Bayesian geometric mean.

**Table 4:** The top 10 signal strengths of AEs of FF/UMEC/VI compared with FF/VI at the PT level.

SOC	PT	*n*	ROR (95%CI)	$\mathrm{PRR}\;(\chi^{2})$
Renal and urinary disorders	Urinary retention	157	8.60 (4.22, 17.50)	8.56 (51.01)
	Bronchospasm paradoxical	93	6.78 (2.97, 15.48)	6.76 (27.73)
	Pollakiuria	55	2.67(1.32, 5.40)	2.67 (8.06)
Gastrointestinal disorders	Lip swelling	30	2.62 (1.02, 6.75)	2.62 (4.29)
	Constipation	84	3.34 (1.78, 6.26)	3.33 (15.90)
	Abdominal discomfort	49	2.38 (1.17, 4.84)	2.38 (6.06)
Investigations	Blood pressure decreased	18	7.86 (1.05, 58.86)	7.85 (5.67)
Nervous system disorders	Taste disorder	63	3.06 (1.52, 6.15)	3.05 (10.89)
Eye disorders	Vision blurred	145	3.02 (1.91, 4.78)	3.01 (24.74)
Infections and infestations	Urinary tract infection	74	2.15 (1.24, 3.75)	2.15 (7.71)

Abbreviations: FF, fluticasone furoate; UMEC, umeclidinium; VI, vilanterol; SOC, system organ class; PT, preferred terms; ROR, reporting odds ratio; CI, confidence interval; PRR, proportional reporting ratio; *χ*^2^, chi-squared.

## Data Availability

The data presented in this study are available in FDA Adverse Event Reporting System at https://www.fda.gov/drugs/fdas-adverse-event-reporting-system-faers/fda-adverse-event-reporting-system-faers-public-dashboard (accessed on 17 December 2024). These data were derived from the following resources available in the public domain: https://www.fda.gov/ (accessed on 17 December 2024).

## Supplementary Materials

The following supporting information can be downloaded at https://www.mdpi.com/article/10.3390/pharmacy13060178/s1, Table S1: FF/UMEC/VI AE signal strength vs. other drugs ranked by SOC frequency; Table S2: Top 30 FF/UMEC/VI AE Ranked by Frequency vs. Other Drugs at PT Level; Table S3: Disproportionality analysis of general, administration site, musculoskeletal, and connective tissue disorders between FF/UMEC/VI and FF/VI.

## Abbreviations

The following abbreviations are used in this manuscript:

AE	Adverse Event
ARR	Absolute Risk Reduction
CI	Confidence Interval
COPD	Chronic Obstructive Pulmonary Disease
DEMO	Demographic/administrative file
DRUG	Drug information file
EBGM	Empirical Bayesian Geometric Mean
FAERS	FDA Adverse Event Reporting System
FEV1	Forced Expiratory Volume in 1 s
FF	Fluticasone Furoate
IC	Information Component
ICS	Inhaled Corticosteroid
IQR	Inter Quartile Range
LABA	Long-Acting Beta2 Agonist
LAMA	Long-Acting Muscarinic Antagonist
MedDRA	Medical Dictionary for Regulatory Activities
PRR	Proportional Reporting Ratio
PT	Preferred Term
PS	Primary Suspect
REAC	Reaction file
ROR	Reporting Odds Ratio
SOC	System Organ Class
SGRQ	St. George’s Respiratory Questionnaire
TTO	Time-To-Onset
UMEC	Umeclidinium
VI	Vilanterol
