# Supplementary material for: Mining of Adverse Event Signals Associated with Fluticasone Furoate/Umeclidinium/Vilanterol Triple Therapy: A Post-Marketing Analysis Based on FAERS
Source: Pharmacy (Basel). 2025 Dec 10;13(6):178. doi: 10.3390/pharmacy13060178 (PMC12737015; doi:10.3390/pharmacy13060178)
Supplement: Supplementary file 1 [file pharmacy-13-00178-s001.zip › pharmacy-3975620-Supplementary.pdf]

Supplementary Materials

Mining of Adverse Event Signals Associated with Fluticasone Furoate/Umeclidinium/Vilanterol Triple Therapy: A Post-Marketing Analysis Based on FAERS

Jiajun Chen [1], Ying Qiao [2], Gaoxing Qiao [3], Xiaocan Jia [2,*] and Jicun Zhu [3,*]

[1] Department of Medical Services, The First Affiliated Hospital of Zhengzhou University, Zhengzhou 450052, China; fccchenjiajun@zzu.edu.cn
[2] Department of Epidemiology and Biostatistics, College of Public Health, Zhengzhou University, Zhengzhou 450001, China; ClyerQY@163.com
[3] Department of Pharmacy, The First Affiliated Hospital of Zhengzhou University, Zhengzhou 450052, China; fccqiaogx@zzu.edu.cn
* Correspondence: jxc@zzu.edu.cn (X.J.); jicunzhu1101@zzu.edu.cn (J.Z.)

Table S1. FF/UMEC/VI AE signal strength vs. other drugs ranked by SOC frequency.

SOC	*n*	ROR(95 % CI)	PRR (χ^2)	IC (IC025)	EBGM (EBGM05)
General disorders and administration site conditions	5,215	0.81 (0.79, 0.83)	0.84 (198.86)	0.26 (0.3)	0.84 (0.82)
Infections and infestations	2,142	1.09 (1.04, 1.13)	1.08 (13.49)	0.11 (0.05)	1.08 (1.04)
Gastrointestinal disorders	1,547	0.54 (0.51, 0.57)	0.56 (573.31)	0.83 (0.91)	0.56 (0.54)
Nervous system disorders	1,439	0.54 (0.52, 0.57)	0.56 (526.82)	0.83 (0.91)	0.56 (0.54)
Surgical and medical procedures	1,210	2.36 (2.23, 2.5)	2.31 (913.4)	1.21 (1.12)	2.31 (2.2)
Investigations	804	0.38 (0.35, 0.41)	0.39 (796.6)	1.34 (1.45)	0.39 (0.37)
Musculoskeletal and connective tissue disorders	776	0.42 (0.39, 0.45)	0.43 (611.29)	1.21 (1.32)	0.43 (0.41)
Eye disorders	666	0.97 (0.9, 1.05)	0.97 (0.45)	0.04 (0.15)	0.97 (0.91)
Cardiac disorders	649	0.95 (0.88, 1.02)	0.95 (1.86)	0.08 (0.19)	0.95 (0.89)
Psychiatric disorders	549	0.28 (0.26, 0.3)	0.29 (999.66)	1.78 (1.9)	0.29 (0.27)
Renal and urinary disorders	533	0.82 (0.75, 0.89)	0.82 (21.45)	0.29 (0.41)	0.82 (0.76)
Neoplasms benign, malignant and unspecified (incl cysts and polyps)	518	0.37 (0.34, 0.41)	0.38 (533.6)	1.38 (1.51)	0.38 (0.36)
Skin and subcutaneous tissue disorders	467	0.23 (0.21, 0.26)	0.24 (1156.84)	2.03 (2.17)	0.24 (0.23)
Vascular disorders	241	0.37 (0.32, 0.42)	0.37 (259.96)	1.42 (1.61)	0.37 (0.34)
Immune system disorders	193	0.47 (0.41, 0.55)	0.48 (112.15)	1.07 (1.28)	0.48 (0.42)
Social circumstances	190	1.13 (0.98, 1.31)	1.13 (2.99)	0.18 (0.03)	1.13 (1.01)
Metabolism and nutrition disorders	169	0.25 (0.21, 0.29)	0.25 (386.34)	2 (2.22)	0.25 (0.22)
Ear and labyrinth disorders	92	0.64 (0.52, 0.78)	0.64 (18.71)	0.64 (0.94)	0.64 (0.54)
Reproductive system and breast disorders	59	0.28 (0.22, 0.36)	0.28 (109.82)	1.83 (2.21)	0.28 (0.23)
Blood and lymphatic system disorders	46	0.07 (0.06, 0.1)	0.08 (525)	3.71 (4.14)	0.08 (0.06)

SOC	*n*	ROR(95 % CI)	PRR (χ^2)	IC (IC025)	EBGM (EBGM05)
Hepatobiliary disorders	23	0.08 (0.05, 0.12)	0.08 (249.66)	3.66 (4.25)	0.08 (0.06)
Endocrine disorders	23	0.25 (0.16, 0.37)	0.25 (53.21)	2.02 (2.61)	0.25 (0.18)
Congenital, familial and genetic disorders	2	0.02 (0.01, 0.09)	0.02 (89.15)	5.54 (7.2)	0.02 (0.01)
Pregnancy, puerperium and perinatal conditions	2	0.02 (0, 0.07)	0.02 (116.23)	5.9 (7.57)	0.02 (0.01)

Abbreviations: FF, fluticasone furoate; UMEC, umeclidinium; VI, vilanterol; SOC, system organ class; *n*, number of cases; ROR, reporting odds ratio; CI, confidence interval; PRR, proportional reporting ratio; χ^2, chi-squared; IC, information component; EBGM, empirical Bayesian geometric mean.

Table S2. Top 30 FF/UMEC/VI AE Ranked by Frequency vs. Other Drugs at PT Level.

SOC	PT	*n*	ROR (95% CI)	PRR (χ^2)	IC (IC025)	EBGM (EBGM05)
Respiratory, thoracic and mediastinal disorders	Dyspnoe	2139	7.67 (7.34, 8.02)	7.27 (11530.5)	2.85 (2.78)	7.2 (6.94)
Respiratory, thoracic and mediastinal disorders	Cough	892	5.45 (5.09, 5.82)	5.33 (3129.01)	2.41 (2.31)	5.3 (5.01)
Respiratory, thoracic and mediastinal disorders	Dysphonia	639	21.07 (19.46, 22.81)	20.71 (11620.28)	4.33 (4.21)	20.09 (18.8)
Infections and infestations	Pneumonia	542	3.11 (2.86, 3.39)	3.08 (761.2)	1.62 (1.49)	3.07 (2.86)
Respiratory, thoracic and mediastinal disorders	Chronic obstructive pulmonary disease	425	18.39 (16.69, 20.27)	18.18 (6715.6)	4.15 (4)	17.71 (16.33)
Infections and infestations	Candida infection	264	24.86 (21.97, 28.12)	24.68 (5777.46)	4.57 (4.39)	23.8 (21.47)
Respiratory, thoracic and mediastinal disorders	Oropharyngeal pain	222	4.18 (3.66, 4.77)	4.16 (529.32)	2.05 (1.85)	4.14 (3.7)
Respiratory, thoracic and mediastinal disorders	Productive cough	185	6.08 (5.26, 7.03)	6.05 (773.94)	2.59 (2.37)	6.01 (5.32)
General disorders and administration site conditions	Chest discomfort	183	3.38 (2.92, 3.91)	3.37 (303.09)	1.75 (1.53)	3.35 (2.97)
Neoplasms benign, malignant and unspecified (incl cysts and polyps)	Lung neoplasm malignant	167	4.63 (3.98, 5.39)	4.61 (469.84)	2.2 (1.97)	4.59 (4.04)
Renal and urinary disorders	Urinary retention	157	9.68 (8.26, 11.33)	9.64 (1197.82)	3.25 (3.02)	9.51 (8.33)
Respiratory, thoracic and mediastinal disorders	Wheezing	153	3.98 (3.39, 4.66)	3.96 (337.42)	1.98 (1.75)	3.95 (3.45)
Respiratory, thoracic and mediastinal disorders	Throat irritation	136	5.87 (4.96, 6.95)	5.85 (542.48)	2.54 (2.29)	5.81 (5.04)
Infections and infestations	Oral candidiasis	126	19.16 (16.04, 22.88)	19.09 (2097.97)	4.21 (3.95)	18.57 (16)
Cardiac disorders	Myocardial infarction	118	2.38 (1.99, 2.85)	2.38 (93.88)	1.25 (0.98)	2.37 (2.04)
Investigations	Oxygen saturation decreased	117	3.13 (2.61, 3.75)	3.12 (167.85)	1.64 (1.37)	3.11 (2.67)
Respiratory, thoracic and mediastinal disorders	Lung disorder	108	3.97 (3.29, 4.8)	3.97 (238.25)	1.98 (1.7)	3.95 (3.37)
Respiratory, thoracic and mediastinal disorders	Rhinorrhoea	108	2.83 (2.34, 3.42)	2.82 (126.65)	1.49 (1.22)	2.81 (2.4)
Respiratory, thoracic and mediastinal disorders	Aphonia	103	12.97 (10.67, 15.77)	12.93 (1112.09)	3.67 (3.38)	12.7 (10.78)
Gastrointestinal disorders	Dry mouth	100	2.6 (2.14, 3.17)	2.6 (97.96)	1.37 (1.09)	2.59 (2.2)
Renal and urinary disorders	Dysuria	93	5.32 (4.34, 6.53)	5.31 (322.95)	2.4 (2.1)	5.28 (4.45)
Respiratory, thoracic and mediastinal disorders	Choking	86	8.46 (6.83, 10.46)	8.44 (556.73)	3.06 (2.75)	8.34 (6.98)
General disorders and administration site conditions	Secretion discharge	69	8.78 (6.92, 11.14)	8.77 (468.45)	3.11 (2.77)	8.66 (7.1)
Respiratory, thoracic and mediastinal disorders	Dyspnoea exertional	68	2.87 (2.26, 3.64)	2.87 (82.42)	1.52 (1.17)	2.86 (2.34)
Psychiatric disorders	Nervousness	63	3 (2.34, 3.85)	3 (83.68)	1.58 (1.22)	2.99 (2.43)
Nervous system disorders	Taste disorder	63	2.89 (2.26, 3.7)	2.89 (77.39)	1.53 (1.16)	2.88 (2.34)

SOC	PT	n	ROR (95% CI)	PRR (χ^2)	IC (IC025)	EBGM (EBGM05)
Cardiac disorders	Cardiac failure congestive	57	2.83 (2.18, 3.67)	2.83 (67.05)	1.5 (1.12)	2.82 (2.27)
Gastrointestinal disorders	Oral pain	55	4.73 (3.63, 6.17)	4.72 (160.26)	2.23 (1.84)	4.7 (3.76)
Renal and urinary disorders	Pollakiuria	55	2.74 (2.1, 3.57)	2.74 (60.32)	1.45 (1.06)	2.73 (2.19)
Gastrointestinal disorders	Retching	53	5.3 (4.04, 6.94)	5.29 (182.89)	2.39 (2)	5.25 (4.19)
Respiratory, thoracic and mediastinal disorders	Dyspnoea	2139	7.67 (7.34, 8.02)	7.27 (11530.5)	2.85 (2.78)	7.2 (6.94)
Respiratory, thoracic and mediastinal disorders	Cough	892	5.45 (5.09, 5.82)	5.33 (3129.01)	2.41 (2.31)	5.3 (5.01)
Respiratory, thoracic and mediastinal disorders	Dysphonia	639	21.07 (19.46, 22.81)	20.71 (11620.28)	4.33 (4.21)	20.09 (18.8)
Infections and infestations	Pneumonia	542	3.11 (2.86, 3.39)	3.08 (761.2)	1.62 (1.49)	3.07 (2.86)
Respiratory, thoracic and mediastinal disorders	Chronic obstructive pulmonary disease	425	18.39 (16.69, 20.27)	18.18 (6715.6)	4.15 (4)	17.71 (16.33)
Infections and infestations	Candida infection	264	24.86 (21.97, 28.12)	24.68 (5777.46)	4.57 (4.39)	23.8 (21.47)
Respiratory, thoracic and mediastinal disorders	Oropharyngeal pain	222	4.18 (3.66, 4.77)	4.16 (529.32)	2.05 (1.85)	4.14 (3.7)
Respiratory, thoracic and mediastinal disorders	Productive cough	185	6.08 (5.26, 7.03)	6.05 (773.94)	2.59 (2.37)	6.01 (5.32)
General disorders and administration site conditions	Chest discomfort	183	3.38 (2.92, 3.91)	3.37 (303.09)	1.75 (1.53)	3.35 (2.97)
Neoplasms benign, malignant and unspecified (incl cysts and polyps)	Lung neoplasm malignant	167	4.63 (3.98, 5.39)	4.61 (469.84)	2.2 (1.97)	4.59 (4.04)
Renal and urinary disorders	Urinary retention	157	9.68 (8.26, 11.33)	9.64 (1197.82)	3.25 (3.02)	9.51 (8.33)
Respiratory, thoracic and mediastinal disorders	Wheezing	153	3.98 (3.39, 4.66)	3.96 (337.42)	1.98 (1.75)	3.95 (3.45)
Respiratory, thoracic and mediastinal disorders	Throat irritation	136	5.87 (4.96, 6.95)	5.85 (542.48)	2.54 (2.29)	5.81 (5.04)
Infections and infestations	Oral candidiasis	126	19.16 (16.04, 22.88)	19.09 (2097.97)	4.21 (3.95)	18.57 (16)
Cardiac disorders	Myocardial infarction	118	2.38 (1.99, 2.85)	2.38 (93.88)	1.25 (0.98)	2.37 (2.04)
Investigations	Oxygen saturation decreased	117	3.13 (2.61, 3.75)	3.12 (167.85)	1.64 (1.37)	3.11 (2.67)
Respiratory, thoracic and mediastinal disorders	Lung disorder	108	3.97 (3.29, 4.8)	3.97 (238.25)	1.98 (1.7)	3.95 (3.37)
Respiratory, thoracic and mediastinal disorders	Rhinorrhoea	108	2.83 (2.34, 3.42)	2.82 (126.65)	1.49 (1.22)	2.81 (2.4)
Respiratory, thoracic and mediastinal disorders	Aphonia	103	12.97 (10.67, 15.77)	12.93 (1112.09)	3.67 (3.38)	12.7 (10.78)
Gastrointestinal disorders	Dry mouth	100	2.6 (2.14, 3.17)	2.6 (97.96)	1.37 (1.09)	2.59 (2.2)
Renal and urinary disorders	Dysuria	93	5.32 (4.34, 6.53)	5.31 (322.95)	2.4 (2.1)	5.28 (4.45)
Respiratory, thoracic and mediastinal disorders	Choking	86	8.46 (6.83, 10.46)	8.44 (556.73)	3.06 (2.75)	8.34 (6.98)
General disorders and administration site conditions	Secretion discharge	69	8.78 (6.92, 11.14)	8.77 (468.45)	3.11 (2.77)	8.66 (7.1)

SOC	PT	*n*	ROR (95% CI)	PRR (χ^2)	IC (IC025)	EBGM (EBGM05)
Respiratory, thoracic and mediastinal disorders	Dyspnoea exertional	68	2.87 (2.26, 3.64)	2.87 (82.42)	1.52 (1.17)	2.86 (2.34)
Psychiatric disorders	Nervousness	63	3 (2.34, 3.85)	3 (83.68)	1.58 (1.22)	2.99 (2.43)
Nervous system disorders	Taste disorder	63	2.89 (2.26, 3.7)	2.89 (77.39)	1.53 (1.16)	2.88 (2.34)
Cardiac disorders	Cardiac failure congestive	57	2.83 (2.18, 3.67)	2.83 (67.05)	1.5 (1.12)	2.82 (2.27)
Gastrointestinal disorders	Oral pain	55	4.73 (3.63, 6.17)	4.72 (160.26)	2.23 (1.84)	4.7 (3.76)
Renal and urinary disorders	Pollakiuria	55	2.74 (2.1, 3.57)	2.74 (60.32)	1.45 (1.06)	2.73 (2.19)
Gastrointestinal disorders	Retching	53	5.3 (4.04, 6.94)	5.29 (182.89)	2.39 (2)	5.25 (4.19)
Respiratory, thoracic and mediastinal disorders	Dry mouth	100	7.67 (7.34, 8.02)	7.27 (11530.5)	2.85 (2.78)	7.2 (6.94)
Respiratory, thoracic and mediastinal disorders	Dysuria	93	5.45 (5.09, 5.82)	5.33 (3129.01)	2.41 (2.31)	5.3 (5.01)
Respiratory, thoracic and mediastinal disorders	Choking	86	21.07 (19.46, 22.81)	20.71 (11620.28)	4.33 (4.21)	20.09 (18.8)
Infections and infestations	Secretion discharge	69	3.11 (2.86, 3.39)	3.08 (761.2)	1.62 (1.49)	3.07 (2.86)
Respiratory, thoracic and mediastinal disorders	Dyspnoea exertional	68	18.39 (16.69, 20.27)	18.18 (6715.6)	4.15 (4)	17.71 (16.33)
Infections and infestations	Nervousness	63	24.86 (21.97, 28.12)	24.68 (5777.46)	4.57 (4.39)	23.8 (21.47)
Respiratory, thoracic and mediastinal disorders	Taste disorder	63	4.18 (3.66, 4.77)	4.16 (529.32)	2.05 (1.85)	4.14 (3.7)
Respiratory, thoracic and mediastinal disorders	Cardiac failure congestive	57	6.08 (5.26, 7.03)	6.05 (773.94)	2.59 (2.37)	6.01 (5.32)
General disorders and administration site conditions	Oral pain	55	3.38 (2.92, 3.91)	3.37 (303.09)	1.75 (1.53)	3.35 (2.97)
Neoplasms benign, malignant and unspecified (incl cysts and polyps)	Pollakiuria	55	4.63 (3.98, 5.39)	4.61 (469.84)	2.2 (1.97)	4.59 (4.04)
Renal and urinary disorders	Retching	53	9.68 (8.26, 11.33)	9.64 (1197.82)	3.25 (3.02)	9.51 (8.33)

Abbreviations: FF, fluticasone furoate; UMEC, umeclidinium; VI, vilanterol; PT, preferred terms; SOC, system organ class; *n*, number of cases; ROR, reporting odds ratio; CI, confidence interval; PRR, proportional reporting ratio; χ^2, chi-squared; IC, information component; EBGM, empirical Bayesian geometric mean.

Table S3. Disproportionality analysis of general, administration site, musculoskeletal, and connective tissue disorders between FF/UMEC/VI and FF/VI.

SOC	*n*	ROR（95%CI）	PRR (χ^2)
Respiratory, thoracic and mediastinal disorders	6,567	1.02 (0.97-1.08)	1.02 (1.31)
Injury, poisoning and procedural complications	9,067	0.76 (0.73-0.79)	0.82 (164.42)

Abbreviations: FF, fluticasone furoate; UMEC, umeclidinium; VI, vilanterol; SOC, system organ class; ROR, reporting odds ratio; CI, confidence interval; PRR, proportional reporting ratio; χ^2, chi-squared.
